# Correction: The use of anticoagulants for rodent control in a mixed-use urban environment in Singapore: A controlled interrupted time series analysis

**DOI:** 10.1371/journal.pone.0310871

**Published:** 2024-09-17

**Authors:** The PLOS One Staff

## Notice of Republication

This article was republished on August 27^th^ 2024, to correct an error in the title introduced during the typesetting process. The publisher apologizes for the errors. Please download this article again to view the correct version. The originally published, uncorrected article and the republished, corrected articles are provided here for reference.

## Supporting information

S1 File Originally published, uncorrected article. (PDF)

S2 File Republished, corrected article. (PDF)
