# Supplementary figures and images for: The use of anticoagulants for rodent control in a mixed-use urban environment in Singapore: A controlled interrupted time series analysis
Source: PLoS One. 2022 May 20;17(5):e0267789. doi: 10.1371/journal.pone.0267789 (PMC9122206; doi:10.1371/journal.pone.0267789)

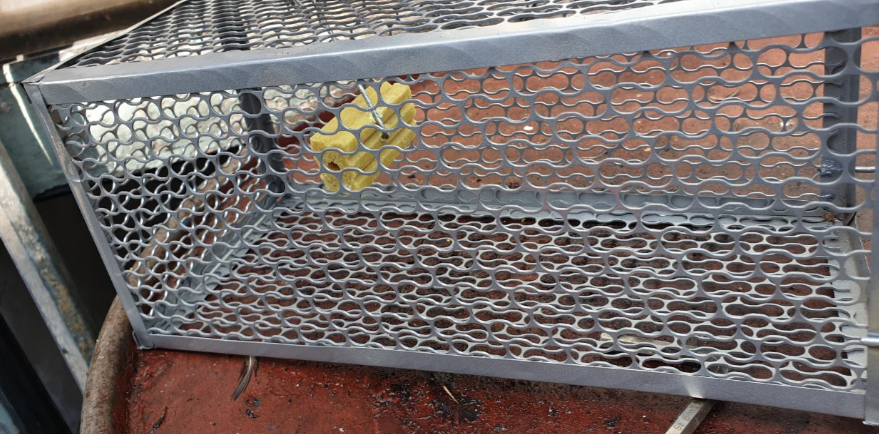

Supplement: S1 Fig — (TIF) [file pone.0267789.s001.tif]

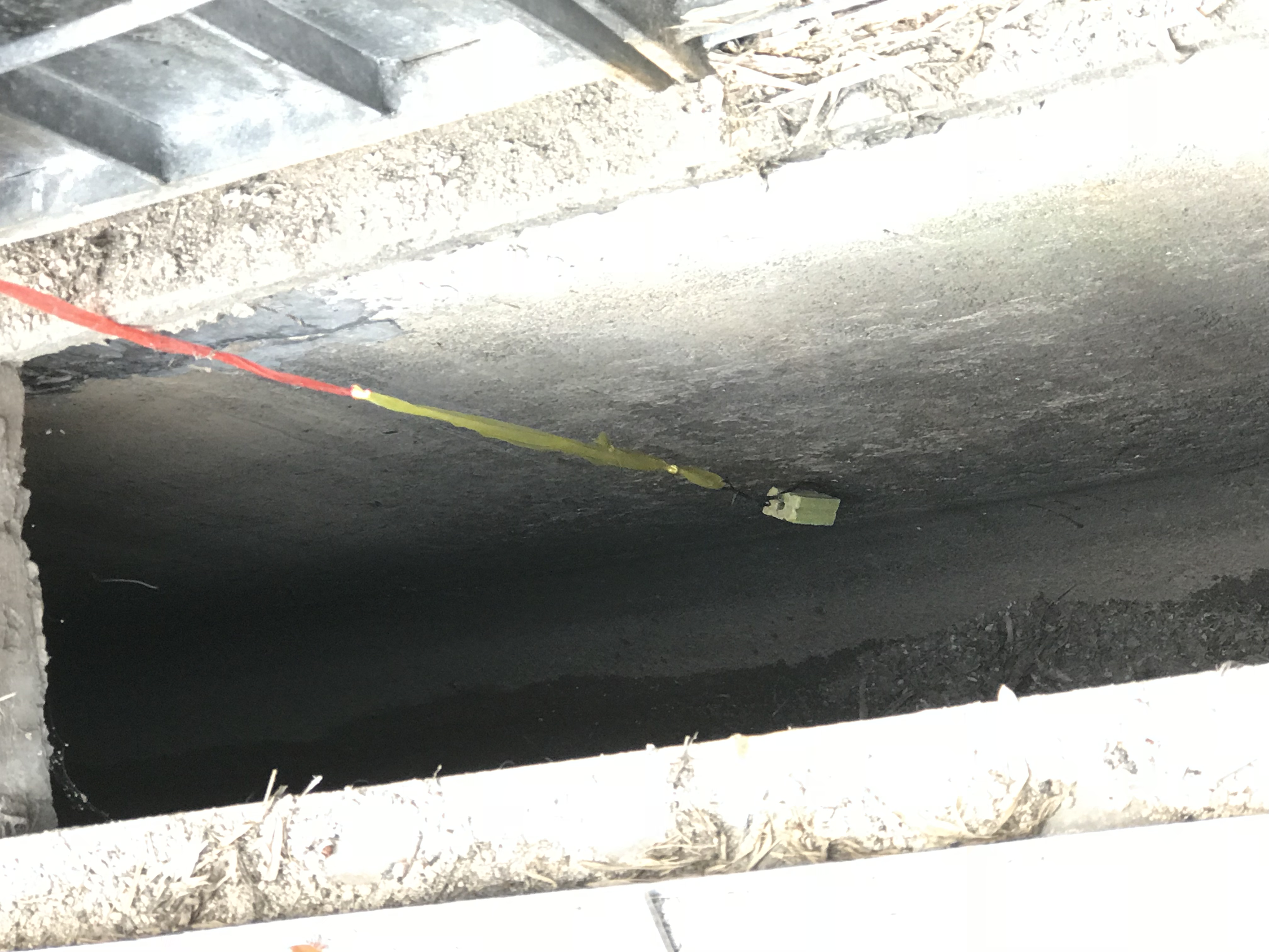

Supplement: S2 Fig — (TIF) [file pone.0267789.s002.tif]
