# Supplementary material for: The use of anticoagulants for rodent control in a mixed-use urban environment in Singapore: A controlled interrupted time series analysis
Source: PLoS One. 2022 May 20;17(5):e0267789. doi: 10.1371/journal.pone.0267789 (PMC9122206; doi:10.1371/journal.pone.0267789)
Supplement: S1 Equation — (DOCX) [file pone.0267789.s003.docx]

**S1 Equation. Equation for outcome measure: number of rodents caught.**

$$log\left( {No. of Rats Caught in Site A}_{t} \right)= \beta_{0}+ \beta_{1} Intervention+\beta_{2} {No. of rats caught in Site B}_{t}+ \beta_{3,4,5,6,7} Deviance {Residual Lag}_{i=1,2,7,13,16}+\beta_{8} Log({No. of Cages in Site A}_{t})$$
