# Supplementary material for: The use of anticoagulants for rodent control in a mixed-use urban environment in Singapore: A controlled interrupted time series analysis
Source: PLoS One. 2022 May 20;17(5):e0267789. doi: 10.1371/journal.pone.0267789 (PMC9122206; doi:10.1371/journal.pone.0267789)
Supplement: S2 Equation — (DOCX) [file pone.0267789.s004.docx]

**S2 Equation. Equation for outcome measure: amount of bait consumed (30g-units).**

$${Bait Consumption in Site A}_{t}= \beta_{0}+ \beta_{1} Intervention+ \beta_{2}{Bait Consumption in Site B}_{t} +\beta_{3, i=1,2,3,4,5} {Day of Week}_{i=1,2,3,4,5}+ \beta_{4,5,6} {Deviance Residual Lag}_{i=3,5,11}+ \beta_{7} Log({No. of Bait Stations and Hanging Baits in Site A}_{t})$$
