# Supplementary material for: The use of anticoagulants for rodent control in a mixed-use urban environment in Singapore: A controlled interrupted time series analysis
Source: PLoS One. 2022 May 20;17(5):e0267789. doi: 10.1371/journal.pone.0267789 (PMC9122206; doi:10.1371/journal.pone.0267789)
Supplement: S3 Equation — (DOCX) [file pone.0267789.s005.docx]

**S3 Equation. Equation for outcome measure: number of marred bait stations.**

$$log\left( No. of Bait Stations with Activty{in Site A}_{t} \right)= \beta_{0}+ \beta_{1} Intervention+\beta_{2} No. of Bait Stations with Activty{in Site B}_{t}+ \beta_{3, i=1,2,3,4,5} {Day of Week}_{i=1,2,3,4,5}+ \beta_{4,5,6,7,8}Deviance {Residual Lag}_{i=2,5,7,8,9}+\beta_{9} Log({No. of Bait Stations in Site A}_{t})$$
