# Supplementary material for: The use of anticoagulants for rodent control in a mixed-use urban environment in Singapore: A controlled interrupted time series analysis
Source: PLoS One. 2022 May 20;17(5):e0267789. doi: 10.1371/journal.pone.0267789 (PMC9122206; doi:10.1371/journal.pone.0267789)
Supplement: S1 Table — (DOCX) [file pone.0267789.s006.docx]

| **Outcome measure** | **Rate Ratio (RR)** | **95% Confidence Intervals** |
| --- | --- | --- |
| **No. of rodents caught** | 0.977 | [0.705, 1.249] |
| **No. of baits consumed** | 1.084 | [0.973, 1.195] |
| **No. of bait stations with activity** | 0.909 | [0.793, 1.025] |

**S1 Table. Pre-intervention trends of outcome measures between the intervention and control sites.**
