# Supplementary material for: The use of anticoagulants for rodent control in a mixed-use urban environment in Singapore: A controlled interrupted time series analysis
Source: PLoS One. 2022 May 20;17(5):e0267789. doi: 10.1371/journal.pone.0267789 (PMC9122206; doi:10.1371/journal.pone.0267789)
Supplement: S2 Table — (DOCX) [file pone.0267789.s007.docx]

| **Outcome measure: Number of rodents caught** | **Incidence Rate Ratio (IRR)** | **95% Confidence Interval** | **P-Value** |
| --- | --- | --- | --- |
| **Post intervention level change** | 0.373 | [0.224, 0.620] | <0.001 |
| **No. of rodents caught in control site** | 1.039 | [0.973, 1.109] | 0.251 |
|  |  |  |  |
| **Outcome measure: Amount of bait consumed (30g-units)** | **Coefficient** | **95% Confidence Interval** | **P-Value** |
| **Post intervention level change** | -25.829 | [-29.855, -21.804] | <0.001 |
| **Bait consumption in control site** | 0.014 | [-0.049, 0.077] | 0.664 |
|  |  |  |  |
| **Outcome measure: Number of marred bait stations** | **Incidence Rate Ratio (IRR)** | **95% Confidence Interval** | **P-Value** |
| **Post intervention level change** | 0.381 | [0.218, 0.665] | <0.005 |
| **No. of marred bait stations in control site** | 0.967 | [0.875, 1.068] | 0.508 |

**S2 Table. Results of the effect of intensive toxic baiting on the number of rodents caught, the amount of baits consumed (30g-units) and the number of marred bait stations.**
