# Supplementary material for: The use of anticoagulants for rodent control in a mixed-use urban environment in Singapore: A controlled interrupted time series analysis
Source: PLoS One. 2022 May 20;17(5):e0267789. doi: 10.1371/journal.pone.0267789 (PMC9122206; doi:10.1371/journal.pone.0267789)
Supplement: Attachment — Submitted filename: Response to Reviewers.docx [file pone.0267789.s009.docx]

**Response to reviewers:**

We thank the reviewers for their comments and suggestions. We have incorporated the majority of them and have provided our responses in blue below for consideration.

**Reviewer #1:**

1. The approach must be reversed, the vaccination plan must be implemented when control strategies fail. Environmental management should be the first option to avoid excessive growth of the rodent population, interaction with humans and the consequent risk of zoonosis due to direct or indirect circulation of pathogens.

Our response: Yes we agree with the reviewer that environmental management should be the first option. We have deleted the following paragraph from our Introduction since our study focus related to population control rather than vaccination:

*“Vaccines against rodent-borne diseases are either unavailable or have limited efficacy. While there are existing vaccines for Leptospirosis, most only provide serogroup-specific immunity and are typically used for short-term prevention in localised outbreaks [10]. The existence of more than 200 serovars of Leptospirosis [11] makes the development of vaccines against multiple strains challenging. A recent study demonstrated the use of inactivated Hantavirus vaccine to prevent the occurrence and progression of HFRS. Results indicated a positive but non-significant inhibitive effect, [12]. Further studies with more subjects are required to ascertain its findings and support vaccination research towards HCPS. Furthermore, the first vaccine for typhus was developed in the early 20^th^ century. It was expensive, hazardous, difficult to produce and no new vaccine has been formulated in the recent years [13].”*

We also amended out Abstract slightly:

“~~As vaccines for rodent-borne diseases have limited efficacy and accessibility,~~ Vector control remains an important strategy in preventing rodent-borne diseases.”

2. Rodents, in addition to transmitting pathogens of zoonotic importance, cause significant damage and economic losses. Food contamination, broken containers and storage bags due to gnawing, as well as fires due to cable bites are some of the main consequences of the action of rodents in high densities. The use of anticoagulants is a strategy to control the population abundance of rodents, which results in a reduction in the transmission of zoonotic diseases, material damage and economic losses.

Our response: Yes we agree with the reviewer and are grateful for these important points. We have now added them into the Introduction (see text in purple):

*“As reservoirs for Hantavirus, aerosolised infected rodents’ droppings and urine are capable of causing Haemorrhagic Fever with Renal Syndrome (HFRS) or Hantavirus Cardio Pulmonary Syndrome (HCPS), depending on the viral strain. Mortality rates of Hantavirus can go up to 12% for HFRS where it is prevalent in Asia and Europe, and 40% for HCPS, which is the prevailing strain in the Americas [5-7]. Parasitic fleas harbouring on rodents are also known to carry Rickettsia typhi which causes murine typhus upon human transmission. The majority of the infections occur in tropical and subtropical countries in Southeast Asia [8, 9]. Rodents, in addition to transmitting pathogens of zoonotic importance, cause significant damage and economic losses. Food contamination, broken containers and storage bags due to gnawing, as well as fires due to cable bites are some of the main consequences of the action of rodents in high densities. The use of anticoagulants is a strategy to control the population abundance of rodents, which results in a reduction in the transmission of zoonotic diseases, material damage and economic losses.”*

3. The experimental site and the control site are markedly different. Yellow shaded areas representing public housing apartment blocks are more abundant at the control site than at the experimental site. These differences generate noise in the results that are not contemplated. Population density data should be included at both sites.

Our response: The size of the yellow areas are indeed different between sites. But the number of residential dwelling units are very similar between sites.

| **Site** | **Total no. of dwelling units** |
| --- | --- |
| Intervention | 550 |
| Control | 517 |

In addition, the pre-intervention trends in both the control and intervention sites were similar and rate ratios of the three outcome measures between sites were non-significant (see our response to Q6 as well). This provides additional support on the similarity between sites for comparability.

We recognize that readers may also raise this point. We have added the following sentence in the Methods section in lines 121-123 *“There was a similar number of residential dwelling units in the intervention (550 units) and the control (517 units) sites.”*

We also inserted a sentence in the Results section in lines 210 to 211 *“There was no difference in the pre-intervention trends between the intervention and control sites (Table S1).”*

4. Intervention: It is necessary to provide more detail on the number of bait units per bait station, to clarify what is referred to as "marred baits" in relation to rodent activity. What type of non-toxic bait was used; were they baits without an active principle?

Our response: We have now provided more details on the number of bait units per bait station, and also improved the definition of “marred baits” in the Methods section. In the pre-intervention period, we used either 1 or 2 bait units per bait station. For the remainder of the study, it was sufficient to use only a single bait unit in every bait station. The non-toxic baits did not have the Bromadiolone active ingredient used in the intervention site. Please refer to the following insertions made in purple text.

*“We used three outcome measures in the study to assess the independent effect of anticoagulant baiting on rodent activity: (i) the number of rodents trapped in cages, (ii) the amount of anticoagulant bait consumed from the cages and bait stations (30g-units) and (iii) the number of marred bait~~s~~ stations. We defined a bait station as being marred if ~~by~~ there was observable evidence of bait consumption on at least one of the bait units within the station.“.*

*“Bell Laboratories CONTRAC BLOX, a second-generation anticoagulant rodenticide [21] containing the active ingredient Bromadiolone (0.005%), weighing approximately 30g per unit, was employed in the cages, bait stations and hanging baits in the treatment site. Bromadiolone is highly toxic and can be lethal to rodents from one day’s feeding [22]. Bromadiolone disrupts the recycling of Vitamin K, preventing blood clotting, resulting in internal bleeding and eventual death [23]. In the control site, Bell Laboratory’s DETEX with Lumitrack baits, which do not contain the active ingredient Bromadiolone, were used.”*

5. Results: Table 1 is confusing. The legend of Table 1 presented in L196 is different from that of L 480, so it is not clear whether the data refer to the amount of bait consumed or the amount of baits placed. In table 1 the name of the phases does not coincide with the name that was made in the text of the ms.

Our response: We noted the reviewer’s request for greater clarity and have now labelled the column headers in Table 1 clearly to reflect that we were referring to the number of cages, hanging baits and bait stations. We also amended the names of the respective phases to be consistent with the manuscript text.

| **Phase** | **Intervention Site** | | | | **Control Site** | | | |
| --- | --- | --- | --- | --- | --- | --- | --- | --- |
|  | **No. of Cages** | **No. of Bait Stations** | **No. of 30g Hanging Baits** | **No. of *R. norvegicus* trapped in cages** | **No. of Cages** | **No. of Bait Stations** | **No. of 30g Hanging Baits** | **No. of *R. norvegicus* trapped in cages** |
| **~~Initial Baseline~~**  **Pre-Intervention**  **(Phase I)** | 39 | 22 | 32 | 95 | 70 | 17 | 8 | 238 |
| **~~De-Ratting~~**  **Intervention**  **(Phase II)** | - | 37 | 60 | - | - | 48 | 65 | - |
| **~~Post-Baseline~~**  **Post-Intervention**  **(Phase III)** | 39 | 22 | 40 | 29 | 70 | 19 | 25 | 142 |
| **Post-Monitoring** | 37 | 18 | 40 | 16 | 70 | 19 | 25 | 85 |

6. The results show the post-intervention changes of the experimental site and the recovery of the rodent population during the monitoring phase; however, the same trend is observed in the control site without having undergone any treatment. It is important to compare the results at both sites to understand the effect of the treatment.

Our response: We would like to clarify that the red dotted lines in Figures 2, 3 and 4 (see below for reference) do not represent the trend in the control group. The red dotted lines represent the theoretical absence of the rodenticide application measures in the treatment site on rodent activity, which already account for the trends in the control site.

| **Figure 2** |  |
| --- | --- |
| **Figure 3** |  |
| **Figure 4** |  |

We did compare the trend for all three outcome measures in the control group and reported the results in Table S1 (see results extracted below for easy reference). Essentially, there was no significant trend in any of the three measures for the control group, therefore providing evidence that the reduction in the measures at the intervention was related to the anticoagulant treatment rather than any changes in the secular trend (as represented by the control site).

| **Outcome measure** | **Incidence Rate Ratio (IRR)** | **95% Confidence Interval** | **p-value** |
| --- | --- | --- | --- |
| **No. of rodents caught in control site** | 1.039 | [0.973, 1.109] | 0.251 |
| **Bait consumption in control site** | 0.014 | [-0.049, 0.077] | 0.664 |
| **No. of marred bait stations in control site** | 0.967 | [0.875, 1.068] | 0.508 |

To improve clarity, we have now added *“There was no significant change in the trend in the control site.”* at the end of the results for each of the three outcome measures.

In addition, we computed the rate ratios (RR) and corresponding CIs for the normalised outcome measures in the pre-intervention period. The normalised rate is the average rate of the outcome measure over the pre-intervention period. The RR is defined as:

$$Rate Ratio \left( RR \right)= \frac{{Normalised rate}_{intervention}}{{Normalised rate}_{control}}$$

| **Outcome measure** | **Rate Ratio (RR)** | **95% Confidence Intervals** |
| --- | --- | --- |
| **No. of rodents caught** | 0.977 | [0.705, 1.249] |
| **No. of baits consumed** | 1.084 | [0.973, 1.195] |
| **No. of bait stations with activity** | 0.909 | [0.793, 1.025] |

The 95% confidence intervals for the RRs overlapped with 1 and were non-significant for all three outcome measures. This supports that both the treatment and control site were similar prior to the intervention.

7.  It is not understood as the effect of anticoagulant bait is not independent of rodent activity. It should clarify this sentence.

Our response: We agree with the reviewer that the effect of anticoagulant bait is not independent of rodent activity. The reviewer may have been referring to the first sentence of the Discussion section. We amended that sentence as follows to improve clarity: “*In this study, we examined the ~~independent~~ effect of anticoagulant rodenticide on rodent activity.*”

8.  There are previous studies in urban environments that show the resilience of rodent populations under chemical control treatments. In all cases they agree that chemical control is only a palliative that without environmental management and health education, rodent control tends to fail. Therefore, I consider that the results obtained are not unknown by the scientific community.

Our response: We agree with the reviewer that previous studies have quantified the effects of chemical control on rodent populations. Studies in South-East Asia are more limited though and we were able to quantify the impact, which can be used to inform the allocation and augmentation of resource plans to improve rodent control. We have made the following amendments to provide a more precise justification for our study:

In the Abstract, we amended the Introduction to *“Studies ~~assessing~~ quantifying the impact of anticoagulant bait use on rodent populations are scarce in tropical settings.”*

In the Introduction, we amended the last sentence as follows: *“In this study, we ~~assessed~~ quantified the impact of intensive anticoagulant use as an urban rodent population control measure, in order to inform the implementation of rodent population control strategies in Singapore.”*
