# Supplementary material for: The use of anticoagulants for rodent control in a mixed-use urban environment in Singapore: A controlled interrupted time series analysis
Source: PLoS One. 2022 May 20;17(5):e0267789. doi: 10.1371/journal.pone.0267789 (PMC9122206; doi:10.1371/journal.pone.0267789)
Supplement: Attachment — Submitted filename: Response to Reviewers.docx [file pone.0267789.s010.docx]

**Response to Reviewers’ Comments**

We are grateful to the reviewer for taking time to assess our manuscript. We have improved our manuscript by implementing the majority of them. We have provided our responses below in blue for the reviewers’ consideration. We have denoted the manuscript text with the use of quotation marks and our amendments in purple font and strikethroughs.

**Reviewer 1**

The explanations and modifications made to the paper by the authors were accepted without inconvenience but pending issues that require attention remain.

We thank the reviewer for suggesting improvements to our manuscript.

**Major Comments:**

1) IRR defined as Incidence Rate Ratio is only described in the annex available only to reviewers. Please include your calculation in the body of the paper (statistical analysis).

We have described the IRR in the Statistical analysis section at lines 202-206:

“The measure of effect for each independent linear term was the incidence rate ratio (IRR) which is the change in the daily proportion of the outcome measures with reference to the mean, associated with a unit change in the corresponding independent exposure. The IRRs were computed by exponentiating the $\beta$ value estimated from the regression analysis. An IRR value below 1.0 denotes a reduction in the outcome for each unit change in the independent variable.”

2) Line 246-247: Since the circulation of pathogens was not specifically evaluated before and after the intervention, it is recommended to also include other problems that can generate such as damage to stored products, fires, etc.

We have noted the reviewer’s recommendation. Although we did not measure other problems such as damage to stored products or fire incidents, we included the following sentence from lines 268-270 to show that these are other possible outcome measures that can be looked at as well.

“Although other rodent activity index measures such as damage to stored products or fire incidents were not evaluated, it could be worthwhile to investigate these in the form of survey questionnaires to shop owners or local managing agents.”

However, we thank the reviewer for a great idea for possible future studies.

3) line 255. It is recommended to review the paper of Fernandez et al. 2007 that provides details in this regard.

We have included the review of this paper in the discussion section at line 276. We also thank the reviewer for the great suggestion of this insightful paper.

4) Line 265: It is also important to consider in order to compare the responses to the use of rodenticides which is the active principle since they present differences in their lethal doses.

We acknowledged the importance of comparing the responses to the use of rodenticides. The other two studies referenced in the discussion both used rodenticides that are anticoagulant based. This is same to what was used in our study. The study done in New Zealand used the same rodenticide bait with the same active ingredient (bromadiolone) as we did, which is a second-generation rodenticide. The other study carried out by United States used diphacinone, which is a first-generation anticoagulant rodenticide and may require higher consumption to cause lethality.

We understand that there are differences in their lethal doses and hence included the sentence below in lines 288-290:

“It is also important to consider the type of anticoagulant used, as anticoagulant rodenticides with different active ingredients will have varying lethal doses that may affect the rate of decline of activity levels.”

5) Line 314: Health education should be considered as a fundamental component of IPM, since without health campaigns it has been shown that all the control strategies that are implemented tend to fail (read the attached bibliography).

We agree with the comment and have included the sentence from lines 348-352:

“Another critical element of IPM would be health and sanitary education. This involves the distribution of educational flyers, publicising awareness posters and community engagement with residents.”

**Reviewer 3**

Despite the importance of the topic of this manuscript, but the authors need to fix several points as follows:

We thank the reviewer for suggesting improvements to our manuscript.

1) The information present in the manuscript are overlapped and need to be rearranged.
- Firstly, the introduction is very short and not properly cover the topic but most required information need are present in the material and methods section. Hence, the authors should transfer the background on the food establishments (lines 74-86) to the introduction. Also, the background on the rodenticide used (lines 114-118) should exist in the introduction.

We agree with the reviewer and have made the following changes:

i) Shifted the background on food establishments to the Introduction at lines 62-71.
ii) Shifted a portion of the background on rodenticides to the Introduction at lines 46-49.

- All possible interpretations of the study findings should exist in the discussion section. E.g. the justification of using the capture-mark-recapture (lines 130-133), the justification of using an interrupted time series analysis (lines 150-153), the justification of inclusion of a control group (lines 154-157), …and so on.

We have added to the discussion on the use of the capture-mark-recapture at lines 321-323:

“The CMR approach was used to estimate the relative rodent abundance and was used as a proxy for rodent activity in this study. This approach was suitable given the difficulty in estimating the true population abundance of the rodent population.”

We have also added to the discussion on the choice of an interrupted time-series analysis at lines 318-319:

“The use of an interrupted time series analysis study design was highly appropriate in evaluating and quantifying the effect of anticoagulant rodenticide control on different measures of rodent activity.”

The justification for the inclusion of the control group was discussed at lines 324-325:

“Any unmeasured secular trends that might have influenced study findings in the intervention site were accounted for with the inclusion of a control site.”

2) The manuscript needs proofreading. Writing style should be formal from the third-person perspective. Do not use we, they, or ours. Please check the whole manuscript.

We have proofread the manuscript and adjusted the writing style accordingly.

3) The legend of Table 1, the location of the study and the duration should be mentioned.

We have included the duration of the study period and specified the study sites in the legend of Table 1 accordingly.

**“Table 1. Number of cages, bait stations and hanging baits deployed in the treatment site (Site A) and control site (Site B) over the study period of 20 weeks.”**
